# Preoperative immunosuppressive therapy might not affect the length of resected bowel in patients receiving ileocolic resection for Crohn’s disease

**DOI:** 10.1007/s00384-025-04927-5

**Published:** 2025-06-05

**Authors:** Matthias Kelm, Michaela Bredl, Anna Widder, Katrin Schoettker, Markus Brand, Alexander Meining, Regina Pistorius, Joachim Reibetanz, Nicolas Schlegel, Christoph-Thomas Germer, Sven Flemming

**Affiliations:** 1 Department for General, Transplant, Vascular and Pediatric Surgery, University Hospital of Wuerzburg, Oberduerrbacher Str. 6, 97080 Würzburg, Germany; 2 Department of Internal Medicine, Division of Gastroenterology, University Hospital of Wuerzburg, Oberduerrbacher Str. 6, 97080 Würzburg, Germany

**Keywords:** Crohn’s disease, Ileocolic resection, Immunosuppressants, Inflammatory bowel disease, Colorectal surgery

## Abstract

**Purpose:**

Rates of surgery remain relevant for localized Crohn’s disease despite the evolution of novel therapies. However, the effect of immunosuppressive medication on the perioperative outcome including the extent of the resection is still inconclusive and needs to be evaluated.

**Methods:**

In a single-center study, all patients who received ileocolic resection due to localized Crohn’s disease were retrospectively assessed and divided into two groups depending on previous treatment (preoperative medication versus therapy-naïve). Outcome parameters included patient characteristics, surgical and histopathological aspects.

**Results:**

Two hundred thirty-seven patients were analyzed of whom 192 patients received medical treatment prior to surgery. Preoperative treatment had neither an effect on the length of the resected specimen (29.4 cm versus 27.1 cm, *p* = 0.27) nor on the postoperative outcome. Only rates of conversion were significantly increased for therapy-naïve patients compared to patients receiving preoperative treatment (15.6% versus 5.7%, *p* = 0.025).

**Conclusion:**

Preoperative medical treatment does not have an effect on the extent of the resected bowel segment in patients suffering from localized Crohn’s disease.

## Background

Increasing incidences of Crohn’s disease (CD) represent an enormous socioeconomic burden for healthcare systems worldwide [1, 2]. While therapeutic strategies are multidisciplinary, specific medical and surgical advancements have resulted in improved treatment options for a large cohort of patients [3]. However, despite novel pharmacological therapies such as biologicals helping to delay surgery, overall surgical rates remain stable over time and current guidelines recommend surgical resection as primary treatment option for localized ileitis based on robust evidence [4, 5]. Indeed, recent data demonstrated advantages for early surgery regarding need for medical therapy and quality of life while medication-related costs are substantial and cannot be neglected [6–9]. While those aspects underline the relevance of surgery for patients suffering from localized CD, medical treatment remains a major factor in multidisciplinary therapies for CD patients [10]. Perioperative pharmacological therapies have been proven to be a valid option for many patients in non-localized disease or postoperative prophylaxis [11]. In line with that, limited data exist on the effect of preoperative medical treatment on the length of the resected bowel segment. This is of great clinical relevance since patients often need multiple resections which might ultimately result in short bowel syndrome; thus, bowel-sparing surgery remains an important issue in daily routine [12]. While several studies addressed this aspect, evidence remains limited and final conclusions are inconclusive without clear recommendations [13–15]. Therefore, to assess the role of preoperative medical therapy on the extent of surgery, we conducted this retrospective study on a homogenous cohort of CD patients receiving ileocecal resection (ICR) due to limited terminal ileitis.

## Material and methods

### Study design

All patients who received ileocolic resection (ICR) due to CD at the Department of Surgery at the University Hospital of Wuerzburg, Germany between 2014 and 2023 were included, and a retrospective analysis was performed. Included patients suffered from localized ileal or ileocolic disease (Montreal classification L1 and L3) with an inflammatory, penetrating and/or stricturing disease behavior (Montreal classification B1-B3). The maximum length of the resection ileum was 40 cm.

### Data acquisition and study population

The length of the resected bowel segment was measured for all patients by the pathologist. Sociodemographic patient characteristics were retrieved from the local database. Evaluated baseline characteristics included age, sex, previous medical and surgical history, and laboratory values (hemoglobin, albumin) to assess potential malnutrition. All patients received MRI and endoscopy preoperatively for diagnostics. Preoperatively, an individual case discussion by a multidisciplinary IBD team including a gastroenterologist, surgeon, pathologist, and radiologist about the indication of surgery was performed. To evaluate the role of preoperative medical therapy on patient outcome, patients were divided into two groups depending on preoperative therapy. Criteria for exclusion were age (< 18 and > 70 years of age), extended resection, emergency surgery, and extraintestinal and multilocalized intestinal disease.

### Outcome

Primary outcome was defined as the amount of the resected bowel segment comparing patients with and without preoperative treatment. Medical therapy was differentiated for glucosteroids (local/topic and systemic), immunomodulators (azathioprine, aminosalicyclic acid), and biologicals (TNFalpha antibodies, monoclonal antibodies) and defined as treatment within 12 weeks prior to surgery. Secondary outcome focused on short-term postoperative morbidity including surgical and non-surgical complications evaluated by the *Comprehensive Complication Index* (CCI), rates of anastomotic leakage, and need for re-admissions. The level of disease severity was evaluated during the histopathological analysis of the resected specimen differentiating between little, moderate, and severe disease severity with score from 1 to 3.

### Statistical analysis

Descriptive data were evaluated and presented as median with range or total numbers with percentages. Differences in patient characteristics as well as primary and secondary endpoints were assessed by a *t*-test, Fisher’s exact test, or ANOVA test in accordance with data scale and distribution. For all analyses, a *p*-value of < 0.05 was considered statistically significant. Statistical analyses were performed using SPSS statistics (version 28, IBM, Armonk, USA) for multivariate analysis.

### Ethics approval

The study was conducted according to the guidelines of the Declaration of Helsinki and approved by the Institutional Ethics Committee of University of Wuerzburg, Germany (Approval number: 2022070601). Informed consent to participate was waived by the Ethics Committee of the University of Wuerzburg due to the retrospective design.

## Results

### Patient characteristics

Two hundred thirty-seven patients received ICR between 2014 and 2023 at the Department of Surgery at the University Hospital of Wuerzburg, Germany. Of those, 192 patients received preoperative immunosuppressive medication such as steroids (*n* = 125), immunomodulators (*n* = 68), and biologicals (*n* = 83). Forty-five patients had not received any medical treatment before surgery (therapy naïve). Sociodemographic characteristics were comparable between both groups regarding sex, age, BMI, smoking habits and malnutrition screening (Table 1). In addition, rates of previous surgical resection were comparable between both cohorts.

**Table 1:** Patient characteristics

	**No treatment** **(*n* = 45)**	**Treatment** **(*n* = 192)**	***p*-value**
Sex, *n* (%) Male Female	24 (53.3) 21 (46.6)	103 (53.6) 89 (46.4)	0.97
Age, years, mean (SD)	39.4 (15.0)	37.9 (15.2)	0.56
BMI, mean (SD)	23.81 (4.8)	23.55 (4.5)	0.74
ASA classification, *n* (%) I II III	2 (4.4) 39 (86.7) 4 (8.9)	10 (5.2) 169 (88) 13 (6.8)	1.0 0.80 0.54
Smoking, *n* (%)	15 (33.3)	55 (28.6)	0.82
Laboratory values Hemoglobin (g/dl), mean (SD) Albumin (g/dl), mean (SD)	12.86 (1.8) 4.08 (0.5)	13.15 (1.8) 4.17 (0.5)	0.35 0.28
Previous ileocolic resection, *n* (%)	12 (26.7)	51 (21.5)	0.42
Disease duration, years, mean (SD)	12.5 (13.9)	10.6 (10.5)	0.31
Preoperative medication, *n* (%) None Steroids Immunomodulators Biologicals	45 0 0 0	0 125 (65.1) 68 (35.5) 83 (43.2)	**0.001**

### Primary outcome

Regarding the primary outcome, the length of the resected specimen was not affected by preoperative medical treatment (29.4 cm versus 27.1 cm, *p* = 0.268) (Table 2). Subgroup analysis confirmed that neither local (27.9 cm, *p* = 0.64) nor systemic steroid treatment (26.7, *p* = 0.49) or biologicals (27.7 cm, *p* = 0.54) had an effect on the extent of the resection compared to therapy-naïve patients. In contrary, only in a small subgroup of patients receiving immunomodulators length of the specimen was reduced (*n* = 19; 21.3 cm, *p* = 0.02) (Table 2).

**Table 2:** Surgical and histopathological characteristics

	**No treatment** **(*n* = 45)**	**Treatment** **(*n* = 192)**	***p*-value**
Surgical approach, *n* (%) Laparoscopic Open	20 (44.4) 25 (55.6)	118 (61.5) 74 (38.6)	**0.04**
Conversion, *n* (%)	7 (15.6)	11 (5.7)	**0.05**
Operating time, min	165.16	160.38	0.60
Stoma, *n* (%)	6 (13.3)	24 (12.5)	0.88
Anastomosis, *n* (%) Side-to-side Side-to-end Kono-S End-to-end	17 (37.8) 9 (20.0) 14 (31.1) 1 (2.2)	86 (44.8) 54 (28.1) 34 (17.7) 4 (2.1)	0.28
Length of resected specimen (cm), mean All Local steroids only Systemic Steroids only Immunomodulators only Biologicals only	29.4	27.1 27.9 26.7 21.3 27.7	0.27 0.64 0.49 0.02 0.54
Disease severity, mean	2.3	2.4	0.50

### Postoperative outcome

To evaluate the postoperative outcome of the medical therapy, various parameters were analyzed. While rates of stoma creation (13.3% versus 12.5%, *p* = 0.88) as well as the type of the anastomosis were comparable between both groups, patients without preoperative treatment had higher rates of conversion to open surgery (15.6% versus 5.7%, *p* = 0.025) (Table 2). Furthermore, no differences were identified for anastomotic leakages (6.7% versus 10.4%, *p* = 0.58) and re-admissions (8.9% versus 14.6%, *p* = 0.23) between both groups. In line with that, overall morbidity was comparable between therapy-naïve patients and patients receiving preoperative treatment as measured by the *Comprehensive Complication Index* (CCI) (24.95 versus 19.76, *p* = 0.14). No postoperative 30-day mortality was observed. (Table 3).

**Table 3:** Postoperative outcome

	**No treatment** **(*n* = 45)**	**Treatment** **(*n* = 192)**	***p*-value**
CCI score, mean	24.95	19.76	0.14
Anastomotic leakage, *n* (%)	3 (6.7)	20 (10.4)	0.58
Wound infection, *n* (%)	16 (35.6)	46 (24.0)	0.13
Postoperative ileus, *n* (%)	13 (28.9)	38 (19.8)	0.23
Hospital re-admission, *n* (%)	4 (8.9)	28 (14.6)	0.23
Mortality, *n* (%)	0 (0)	0 (0)	–

## Discussion

CD remains an incurable disease and multidisciplinary treatment strategies challenging. While recent evidence and guidelines implemented surgery as primary treatment option for localized ileocecal disease with long-term superiority over medical therapy [6, 8, 16], robust evidence about the potential beneficial effect of additional immunosuppressive medication on the extent of the resection is limited and heterogenous. However, this remains an important question in daily clinical routine since bowel-sparing surgery is a primary dogma in CD patients. To address this aspect, we performed an analysis of our own large patient cohort focusing on the perioperative impact of medical treatment on surgical specimens and morbidity. Based on our presented data, we did not find any evidence that preoperative immunosuppressive medication influences the extent of the resected specimen.

In this monocentric study, 237 patients who received ileocolic resection were included of which 45 patients were therapy-naïve at the time of surgery. Statistical analysis revealed that preoperative medical treatment had no effect on the length of the resected specimen (29.4 cm versus 27.1 cm, *p* = 0.268) (Table 2). While this effect was confirmed for medications such as steroids and biologicals in subgroup analysis (Table 2), immunomodulators demonstrated a significant benefit to reduce the extent of the resection. However, this was seen in a small cohort (*n* = 19) only and might be explained by a selection bias. While preoperative immunosuppressive medication did not result in increased rates of stoma creation, perioperative morbidity was also not affected by additional medical therapy as evaluated by the CCI score (24.95 versus 19.76, *p* = 0.14). In line with that, rates of anastomotic leakages (6.7% versus 10.4%, *p* = 0.58) and re-admissions (8.9% versus 14.6%, *p* = 0.23) were comparable between both groups of operated patients. However, specific dosages and dates of last application prior to surgery were lacking, thus, limiting the final conclusion about the perioperative safety of the evaluated medication.

An American population-based cohort study demonstrated the often-extensive overall length of resected bowel segments in CD patients [17], thus, underlining the need for bowel-sparing procedures which has been implemented in national and international guidelines. Preoperative medical therapy could potentially decrease the level and amount of inflammation, thus, minimizing the extent of the bowel resection. While Peyrin-Biroulet et al. did not investigate the effect of medications on the length of the resected bowel segment, they showed that only corticosteroids were associated with the time interval between the first and second resection but other medical therapies had no impact [17]. Similarly, several studies demonstrated that the median length resected is the largest at the first surgery with a subsequent decrease during follow-up operations and an observational study identified that the length of the resected bowel segment is significantly longer during emergency operations [18–20]. Regarding the effect on the extent of the intestinal resection, Kamel et al. demonstrated a decreased length of resected specimen for CD patients receiving biologicals preoperatively. However, colonic resections (61.1%) and urgent procedures (17.4%) were also included, thus, limiting a final conclusion [21]. In contrast, a small Belgian study did not find any significant correlation between TNFalpha application and a reduction of the length of the resected bowel segment [15]. This was confirmed by de Groof et al. who demonstrated in a time trend analysis that a more intense medical treatment had no impact on the specimen size [14]. In line with that, Fu et al. investigated this important aspect and did not find an impact of anti-TNF agents on the amount of small bowel resected by comparing two national cohorts before and after the availability of anti-TNF agents [13]. However, the conclusion of this data is limited since the cohorts were included before the year 2010 and medical treatment changed relevantly since then. Therefore, robust evidence on the exact effect of preoperative medical treatment on the extent of the specimen size considering current therapeutics is lacking. In contrast, our study time focused on the years 2014 to 2023 which included novel therapeutic strategies. However, despite the evolution of medical agents over time, no effect of preoperative immunosuppressants on the length of the resected bowel segments could be observed in our cohort, thus, our data is in line with prior results of de Groof and Fu et al. Based on this evidence, preoperative medical treatment can currently not be recommended to reduce the extent of the bowel resection in case of localized disease. Nevertheless, medical therapy is rapidly evolving in IBD and novel medical strategies might have the potential to affect the amount of the resection, thus, a periodic evaluation is advisable.

Our study has relevant limitations including the retrospective design and the single-center design which might result in a selection bias. In addition, certain aspects of the medical disease history such as initial disease extent, time intervals and dosages prior to surgery are lacking which limits the final conclusion on the effect of medications. However, we investigated a large homogenous cohort including only patients suffering from localized Crohn’s disease and receiving an ileocolic resection as standardized procedure, thus, clearly strengthening the results of the study. In addition, our study time from 2014 to 2023 included current pharmacological treatment strategies.

## Conclusion

Since surgery is recommended as primary treatment option comparable to medical therapy for localized ileocolonic disease in current guidelines, the dogma to perform bowel-sparing procedures gains new attention including the potential effect of additional preoperative medical therapy. However, despite the evolution of novel therapeutics over time, there is currently no evidence that preoperative medical treatment can reduce the length of the resected bowel segment. Nevertheless, it has to be kept in mind that the available studies have relevant limitations and no prospective data exists to date. These issues and results underline the complexity of the disease as well as the need for novel multidisciplinary treatment strategies including periodic reevaluation.

## Data Availability

Data is available on request.
